# Human reference microbiome profiles of different body habitats in healthy individuals

**DOI:** 10.3389/fcimb.2025.1478136

**Published:** 2025-02-11

**Authors:** Sujin Oh, Kyoung Un Park

**Affiliations:** ^1^ Department of Laboratory Medicine, Seoul National University College of Medicine, Seoul, Republic of Korea; ^2^ Department of Laboratory Medicine, Seoul National University Bundang Hospital, Seongnam, Republic of Korea

**Keywords:** human reference microbiome, healthy microbiome status, blood microbiome, saliva microbiome, stool microbiome, taxonomic profile

## Abstract

**Introduction:**

This study aimed to establish the human reference microbiome profiles in blood, saliva, and stool of healthy individuals, serving as reference values to identify microbiome alterations in human disease.

**Methods:**

The study population consisted of a reference group of healthy adults and a second group consisting of adults with periodontal disease (PD). Blood, saliva, and stool samples were subjected to 16S rRNA sequencing. Reference intervals of alpha diversity indices were calculated. To reduce the effects of inherent limitations of microbiome data, the taxonomic profiles of the reference group were estimated as log-scaled fold change (logFC) in the abundance of microorganisms between two habitats within the subjects.

**Results:**

For stool and saliva microbiomes, differences in the abundances of Firmicutes, Patescibacteria, and Verrucomicrobia distinguished healthy from PD subjects (95% confidence interval (CI) of logFC: [−0.18, 0.31], [−1.19, −0.34], and [−3.68, −2.90], respectively). Differences in the abundances of Cyanobacteria, Fusobacteria, and Tenericutes in stool and blood microbiome of healthy subjects fell within 95% CI of logFC [−0.38, 0.61], [−4.14, −3.01], and [1.66, 2.77], respectively. In saliva and blood, differences in the abundances of Epsilonbacteraeota, Firmicutes, Fusobacteria, and Proteobacteria could be used as reference values (95% CI of logFC: [−3.67, −2.47], [−0.35, 0.49], [−4.59, −3.26], and [−1.20, 0.07], respectively).

**Discussion:**

As the reference microbiome profiles could discern healthy subjects and individuals with PD, a relatively mild disease state, they can be applied as reference values representing the healthy status of the microbiome and for screening of disease states, preferably in preclinical stages.

## 1 Introduction

The human microbiome is the community of microorganisms, including bacteria, viruses, and fungi, that occupy various habitats of the body (Consortium HMJRS et al., 2010). The total number of microbial genomes outnumbers the number of human genomes by several orders of magnitude (Gill et al., 2006). Therefore, the human genetic landscape is now thought of as a collection of human and microbial genomes, and human metabolic functions are viewed as a mixture of human and microbial traits (Turnbaugh et al., 2007). With the advent of high-throughput next-generation sequencing, metagenomics allows researchers to study the profiles of entire microbiome communities without being limited to the identification of specific microbial species (Jovel et al., 2016).

Beyond understanding the role of microbial communities in the various environments of the human body, current microbiome studies aim to apply the microbiome to the diagnosis of human diseases. Most studies have sought disease-associated microbiomes, which could potentially be used as novel diagnostic markers and for early disease management. The best studied of these is the gut microbiome, including research on its involvement in the pathogenesis of gastrointestinal diseases, such as inflammatory bowel disease (Manichanh et al., 2012), and several types of cancer, including colorectal, gastric, and liver cancer (Meng et al., 2018).

Microbial dysbiosis in the oral cavity has been implicated in the development of many non-communicable diseases. Several studies have shown that periodontal disease (PD) is closely related to the onset of systemic diseases, including cardiovascular disease (Persson and Persson, 2008), Alzheimer’s disease (Dioguardi et al., 2020), and certain types of cancer (Dizdar et al., 2017).

Blood in the closed circulatory system has been considered a sterile environment, and it was commonly believed that microbes were present in the blood only in sepsis. Over the past decade, however, the blood microbiome has been described in the context of various diseases, including cardiovascular events (Rajendhran et al., 2013), liver cirrhosis (Santiago et al., 2016), and diabetes (Amar et al., 2011), without any clinical evidence of infection. Numerous microorganisms have also been detected in healthy blood donors (Rajendhran et al., 2013; Païssé et al., 2016; Santiago et al., 2016).

Although efforts have been made to enable the diagnostic use of microbiome data, a laboratory test result is of little value by itself unless it is reported with the appropriate information for its interpretation. The information is typically provided in the form of reference intervals or medical decision limits and includes only the values of the reference group and excludes others (Ozarda, 2016). Therefore, characterizing the baseline state of the microbiome in a healthy reference group is an important first step in determining the pathological microbial state.

In this study, we established the human reference microbiome profiles in various body habitats of healthy individuals. The reference microbiome refers to the community of microorganisms in healthy individuals without overt signs of disease, and its composition can be used as the reference status for the detection of microbiome alterations in human disease. Here, we suggested the reference values of the healthy microbiome, representing the baseline status of taxonomic compositions in different body habitats, including blood, saliva, and stool, which could differentiate between healthy and disease states. The study population consisted of two groups: a reference group of healthy adults, and a PD group consisting of adults with PD. We first investigated the microbial diversity and taxonomic profiles of the reference group. Then, we selected a subset as the reference microbiome profile for which the corresponding values for all subjects in the PD group, a relatively mild disease, deviated from that of the reference group. The determination of reference profiles of the microbiome communities in various body habitats will facilitate early detection of changes in the microbiome associated with disease states, preferably in preclinical stages.

## 2 Materials and methods

### 2.1 Study design

Subjects were recruited from among those who visited the dental clinic of Seoul National University Bundang Hospital (SNUBH) and were divided into two groups. The reference group chosen according to predefined criteria consisted of 171 healthy people aged between 18 and 89 years. A total of 339 samples, including 117 blood, 124 saliva, and 98 stool samples, were obtained from this group. Among them, 77 paired samples of blood and saliva were included, while 65 of stool and saliva and 72 of stool and blood did. The PD group consisted of ten subjects with PD in the absence of other diseases. These subjects did not have any symptoms or signs of systemic infection but showed localized periodontal inflammation at the time of sample collection.

Healthy subjects in this study were carefully selected by evaluation for the presence or absence of systemic disease. The purpose of the study and the types of samples required were described to all potential participants. A questionnaire survey with inquiries about the history of diseases, including hypertension, diabetes, chronic infection, liver, kidney, cardiovascular disease, autoimmune disease, and malignant tumors, and history of antibiotic use within 6 months, was conducted for prescreening of potential study participants. In addition, lifestyle factors that could potentially influence the microbiome, such as alcohol consumption and tobacco smoking habits, were collected through questionnaires. Electronic medical records, including routine laboratory test results, and dental charts were also reviewed to ascertain subjects’ medical history and additional clinical characteristics. After further assessment of eligibility, subjects who provided written informed consent were included in the study. Subjects were requested to refrain from oral hygiene activities at least 2 h prior to sample collection. All samples were collected before any dental procedures that could have altered the oral microbiome. The demographic and clinical characteristics of subjects are listed in **Table 1**.

**Table 1:** Clinical information of study participants.

Features			Reference group (N = 171)	PD (N = 10)	P-value
Age (yrs)		Mean ± sd	45.0 ± 14.9	42.8 ± 8.97	0.648
Age (yrs), categorical		N (%)			0.69
	<20		1 (0.6)	0 (0.0)	
	20–40		69 (40.4)	4 (40.0)	
	40–60		80 (46.8)	6 (60.0)	
	60–80		20 (11.7)	0 (0.0)	
	≥80		1 (0.6)	0 (0.0)	
Sex		N (%)			0.339
	M		82 (48.0)	3 (30.0)	
	F		89 (52.0)	7 (70.0)	
BMI (kg/m^2^)^*^		Mean ± sd	25.0 ± 5.1	23.2 ± 5.2	0.089
Alcohol consumption^*^		N (%)			0.048
	Yes		77 (45.3)	8 (80.0)	
	No		93 (54.7)	2 (20.0)	
Smoking habits^*^		N (%)			0.285
	Yes		17 (10.0)	20 (20.0)	
	No		153 (90.0)	80 (80.0)	

PD, periodontal disease; BMI, body mass index.

^*^BMI, alcohol consumption, and smoking habit data were not available for the subset of the reference group; 39, one and one were missing, respectively.

### 2.2 Sample collection and preparation

Blood, saliva, and stool samples were collected from each subject. Venous blood samples were collected under sterile conditions by trained personnel. Specimens were collected from the oral cavity in a uniform manner by one trained investigator to minimize batch effects. Stool samples were collected by the subjects themselves using a sterile spatula, placed in a sterile stool sample container, and stored in a freezer until transport to the lab on ice. After collection, all specimens except stool samples were immediately transported to the laboratory and stored at −70°C until DNA extraction. Microbiome DNA was isolated from each specimen using a QIAamp DNA Microbiome Kit (QIAGEN, Venlo, The Netherlands) according to the manufacturer’s standard protocol.

### 2.3 16S rRNA sequencing

Each sample was prepared according to the Illumina 16S Metagenomic Sequencing Library protocol to amplify the V3 and V4 regions (519F-806R). DNA quality was measured using Qubit dsDNA HS Assay Kits (Thermo Fisher Scientific Inc., Waltham, MA, USA). PCR was performed using KAPA HiFi HotStart ReadyMix PCR kits (Roche, Basel, Switzerland) in accordance with the manufacturer’s instructions. The primer sequences used for PCR amplification were as follows: 519F: 5′-CCTACGGGNGGCWGCAG-3′; 806R: 5′-GACTACHVGGGTATCTAATCC-3′. Libraries were constructed with NextEra XT DNA library preparation kits (Illumina Inc., San Diego, CA, USA) and pooled to a final loading concentration of 8 pM. Next, paired-end (2 × 300 bp) sequencing was performed using the MiSeq platform (Illumina).

### 2.4 16S metagenomics data analysis

The reads were processed using a Divisive Amplicon Denoising Algorithm (DADA) 2-based pipeline conducted within the QIIME2 22.2 platform (Callahan et al., 2016; Bolyen et al., 2019). Briefly, an amplicon sequencing variant (ASV) table was produced by quality-based filtering and trimming, read deduplication, and inference of ASVs, followed by paired-end merging and chimera removal. To correct for artifactual biases, the feature tables were normalized by rarefaction. For alpha diversity analysis, indices such as Shannon’s entropy, Pielou’s evenness, and Simpson’s index were measured. To estimate the dissimilarities between the microbial compositions, beta diversity indices including Bray-Curtis and unweighted UniFrac distance matrices were computed, and permutation multivariate analysis of variance (PERMANOVA) of the diversity matrix was conducted to quantify the strengths of the associations between community composition and variables. For taxonomic analysis of the microbial composition, the sequences were taxonomically classified against the 99% SILVA rRNA taxonomy using a pre-trained scikit-learn naive Bayes machine learning classifier of the q2-feature-classifier plugin (Quast et al., 2012).

For differential abundance (DA) analysis, we conducted ANCOM-BC, which estimates the unknown sampling fractions and corrects for the bias induced by their differences through a log-linear regression model (Lin and Peddada, 2020). We determined the differentially abundant taxa between each of the two body habitats if false discovery rate (FDR) < 0.05 and log-scaled fold change (logFC) values of microbial abundance and their 95% confidence intervals (CIs) were estimated.

### 2.5 Statistical analysis

Statistical analysis and data visualization processes other than those described above were performed using R software (ver. 4.1.2; R Development Core Team, Vienna, Austria). QIIME artifacts were imported into the R environment using the *qiime2R* package and converted into phyloseq objects using the *phyloseq* package (McMurdie and Holmes, 2013). Associations between categorical variables were assessed using either the Chi-square test or Fisher’s exact test, depending on the data distribution. Nonparametric tests were performed using the two-sided Wilcoxon’s rank-sum test. Correlations between diversity indices and continuous variables were estimated through Spearman’s correlation analysis (the Mantel permutation test was used for beta diversity). Reference intervals were calculated as 2.5th and 97.5th percentiles of the values from the reference group. Principal component analysis (PCA) was performed to elaborate on the distinction between the reference and PD groups. Statistical significance was defined as a p-value < 0.05. Otherwise, for analyses involving multiple comparisons, statistical significance was determined using a FDR threshold of < 0.05, corrected using the Benjamini-Horchberg method.

## 3 Results

### 3.1 Characteristics of study population

The reference group consisted of 171 carefully selected healthy subjects based on predefined criteria, while the PD group comprised 10 individuals who, at the time of inclusion, showed localized periodontal inflammation without any symptoms of systemic infection. The characteristics of the study population are presented in **Table 1**. The mean age of the reference group was 45.0 ± 14.9 years (mean ± sd), compared to 42.8 ± 8.97 years in the PD group, with the majority of subjects in both groups aged between 20 and 60 years. In terms of sex distribution, 48.0% (n = 82) of the reference group were male and 52.0% (n = 89) were female, compared to 30.0% (n = 3) male and 70.0% (n = 7) female in the PD group. The average body mass index (BMI) was 25.0 ± 5.1 kg/m² for the reference group and 23.2 ± 5.2 kg/m² for the PD group. Regarding lifestyle habits, 80.0% (n = 8) of the PD group reported alcohol consumption compared to 45.3% (n = 77) in the reference group (*p* = 0.048), whereas prevalence of smoking was 10.0% and 20.0% for healthy subjects and subjects with PD, respectively. Baseline demographics, including age, sex, and BMI, did not differ significantly between the reference and PD groups (*p* > 0.05), indicating well-balanced characteristics for reliable comparison.

### 3.2 Baseline diversity of blood, saliva, and stool microbiomes of reference group

To establish baseline knowledge regarding microbial diversity, we analyzed alpha and beta diversity of the blood, saliva, and stool microbiomes in the reference group, considering variations by sex, age, and additional clinical variables such as BMI, alcohol consumption, and smoking habits. As shown in **Supplementary Figure S1**, sex did not significantly impact the richness or evenness of microbiomes across blood, saliva, and stool. Sex similarly did not contribute to taxonomic dissimilarities measured by beta diversity indices for blood, saliva, and stool, with only 1.6% of variation in Bray-Curtis distances of blood microbiome attributed to sex (*p* = 0.014; **Supplementary Table S1**). Spearman’s correlation was examined to identify any age-derived variations in the alpha and beta diversity of the three habitats. Only the saliva microbiome showed a weak association between age and Bray-Curtis dissimilarity (ρ = 0.08, *p* = 0.01); there were no significant age-associated differences in the other diversity indices (**Supplementary Figure S2**; **Supplementary Table S2**).

BMI associations were similarly examined. For blood and saliva, neither alpha nor beta diversity indices showed significant relationships with BMI. In the stool microbiome, however, richness and evenness declined as BMI increased, although the significance levels varied across indices [*p* = 0.166 (Shannon’s entropy), = 0.046 (Simpson’s index), and = 0.023 (Pielou’s evenness); **Supplementary Figure S2**]. In addition, BMI did not significantly impact beta diversity indices for any habitat (**Supplementary Table S2**). Regarding lifestyle factors, smoking status had not significant effect on either alpha or beta diversity in blood, saliva, or stool microbiomes (**Supplementary Figure S1**; **Supplementary Table S1**). In contrast, subjects who reported alcohol consumption exhibited increased richness and evenness across all microbiomes, though no single habitat showed significant differences consistently across all three alpha diversity indices. For beta diversity, alcohol consumption explained 3.4% of the variation in blood microbiomes by Bray-Curtis distances and 2% by unweighted UniFrac distances (*p* < 0.05; **Supplementary Table S1**).

**Figure 1:**
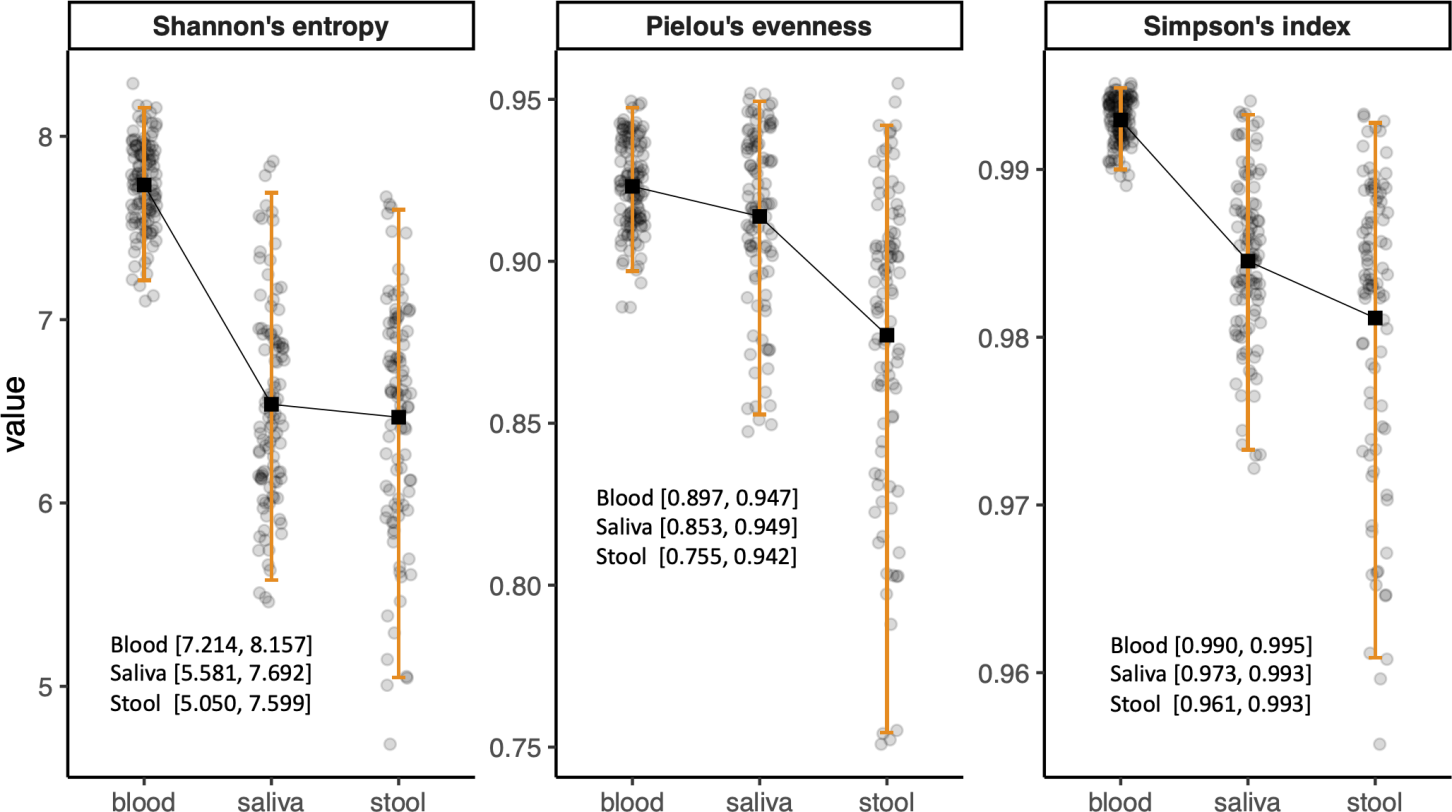
Bar plots showing the reference intervals of alpha diversity indices. The reference intervals were calculated as 2.5th and 97.5th percentiles of the values after outliers were excluded.

Due to the clinical relevance of age- and sex-stratified reference intervals, we evaluated the need for such stratification in microbiome diversity indices. However, as no sex- or age-related variations were found in microbial compositions, the reference intervals for alpha diversity of the microbiome in each habitat were calculated as the central 95% of the reference group with exclusion of only outliers. The reference intervals of blood microbiome diversity were 7.214–8.157, 0.990–0.995, and 0.897–0.947 for Shannon’s entropy, Simpson’s index, and Pielou’s evenness, respectively. The alpha diversities of the saliva microbiome in the reference group were 5.581–7.692, 0.973–0.993, and 0.853–0.949 for Shannon’s entropy, Simpson’s index, and Pielou’s evenness, respectively, whereas those of the stool microbiome were 5.050–7.599, 0.961–0.993, and 0.755–0.942, respectively (**Figure 1**).

### 3.3 Taxonomic profiles of blood, oral, and stool microbiomes of reference group

**Figure 2:**
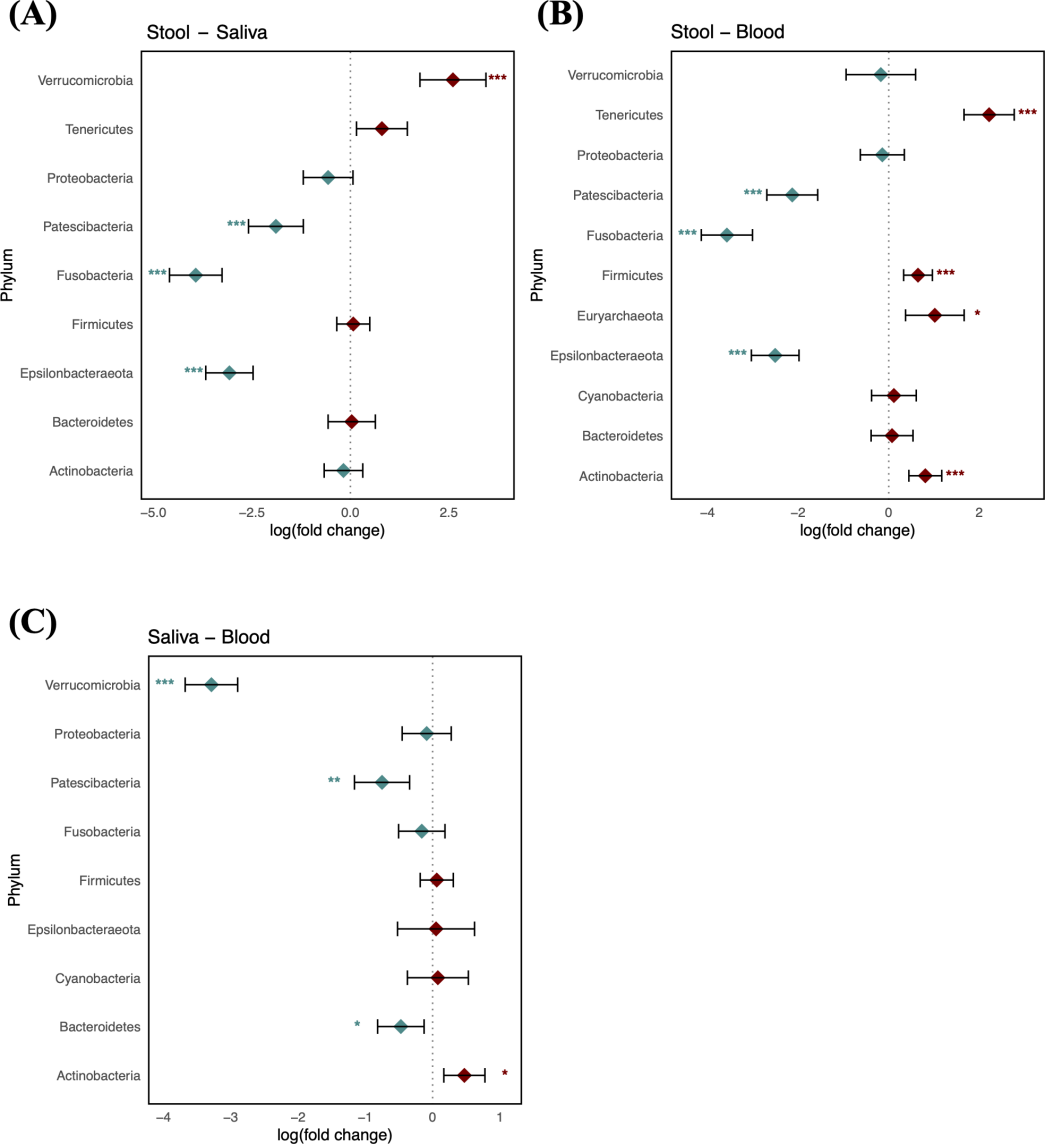
Log-scaled fold change (logFC) and 95% confidence interval (CI) of the abundance of each phylum estimated by differential abundance tests. Positive logFC values indicate a higher abundance in the first sample type listed (e.g., stool in panel A) compared to the second (e.g., saliva in panel A), while negative values indicate a higher abundance in the latter. **(A)** LogFC in stool vs. saliva; **(B)** stool vs. blood; and **(C)** saliva vs. blood. Points represent the estimated logFC for each phylum, with red points indicating positive and blue points indicating negative value. Error bars represent the 95% CI for each estimate. *, false discovery rate (FDR) < 0.05; **, FDR < 0.01; ***, FDR < 0.001, denoting levels of statistical significance in abundance differences.

Taxonomic characterization of microbiomes in blood, saliva, and stool of the reference group was performed through pairwise DA testing at phylum and genus levels. The stool microbiome was characterized by greater abundances of Patescibacteria, Fusobacteria, and Epsilonbacteraeota (logFC: −1.89 [95% CI: −2.58, −1.19], −3.92 [−4.59, −3.26], and −3.07 [−3.67, −2.47], respectively) and by a lower abundance of Verrucomicrobia compared to saliva (logFC: 2.60 [1.76, 3.44]) (**Figure 2A**). The saliva microbiome showed a greater abundance of Actinobacteria, whereas Verrucomicrobia, Patescibacteria, and Bacteroidetes were more abundant in the blood microbiome (logFC: 0.47 [95% CI: 0.17, 0.78], −3.29 [−3.68, −2.90], −0.75 [−1.16, −0.34], and −0.47 [−0.82, −0.13], respectively). The blood microbiome in healthy individuals showed a relative enrichment of Patescibacteria compared to saliva and stool, as well as a higher relative presence of Verrucomicrobia, although its abundance did not significantly differ from that in stool (FDR > 0.05) (**Figures 2B, C**).

**Figure 3:**
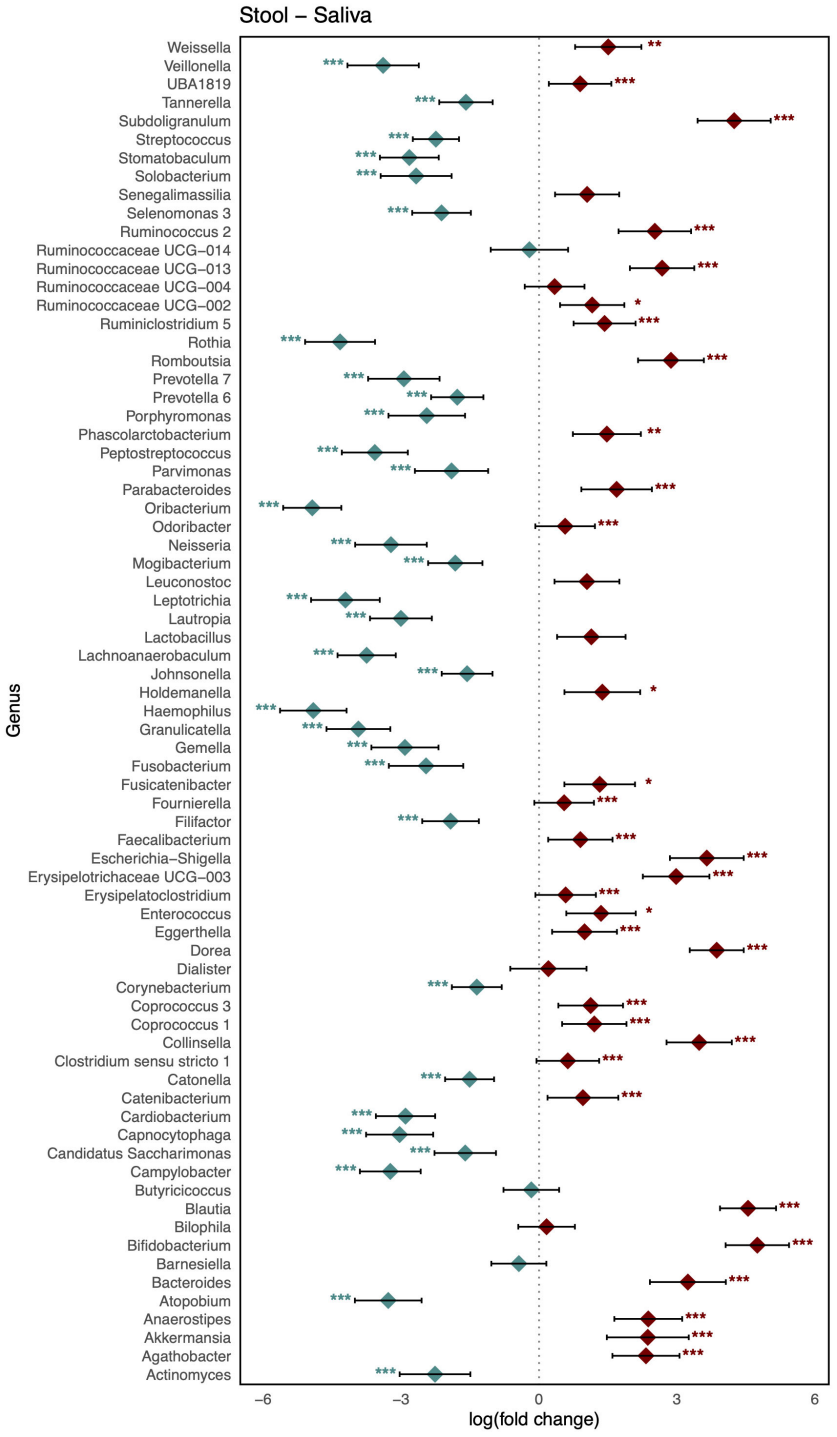
Log-scaled fold change (logFC) and 95% confidence interval (CI) of the abundance of each genus in stool vs. saliva confirmed by differential abundance tests. Positive logFC values indicate a higher abundance in stool compared to saliva, while negative values indicate a higher abundance in the latter. Points represent the estimated logFC for each genus, with red points indicating positive (higher abundance in stool) and blue points indicating negative (higher abundance in saliva) values. Error bars represent the 95% CI for each logFC estimate. *, false discovery rate (FDR) < 0.05; **, FDR < 0.01; ***, FDR < 0.001, denoting levels of statistical significance in abundance differences.

**Figure 4:**
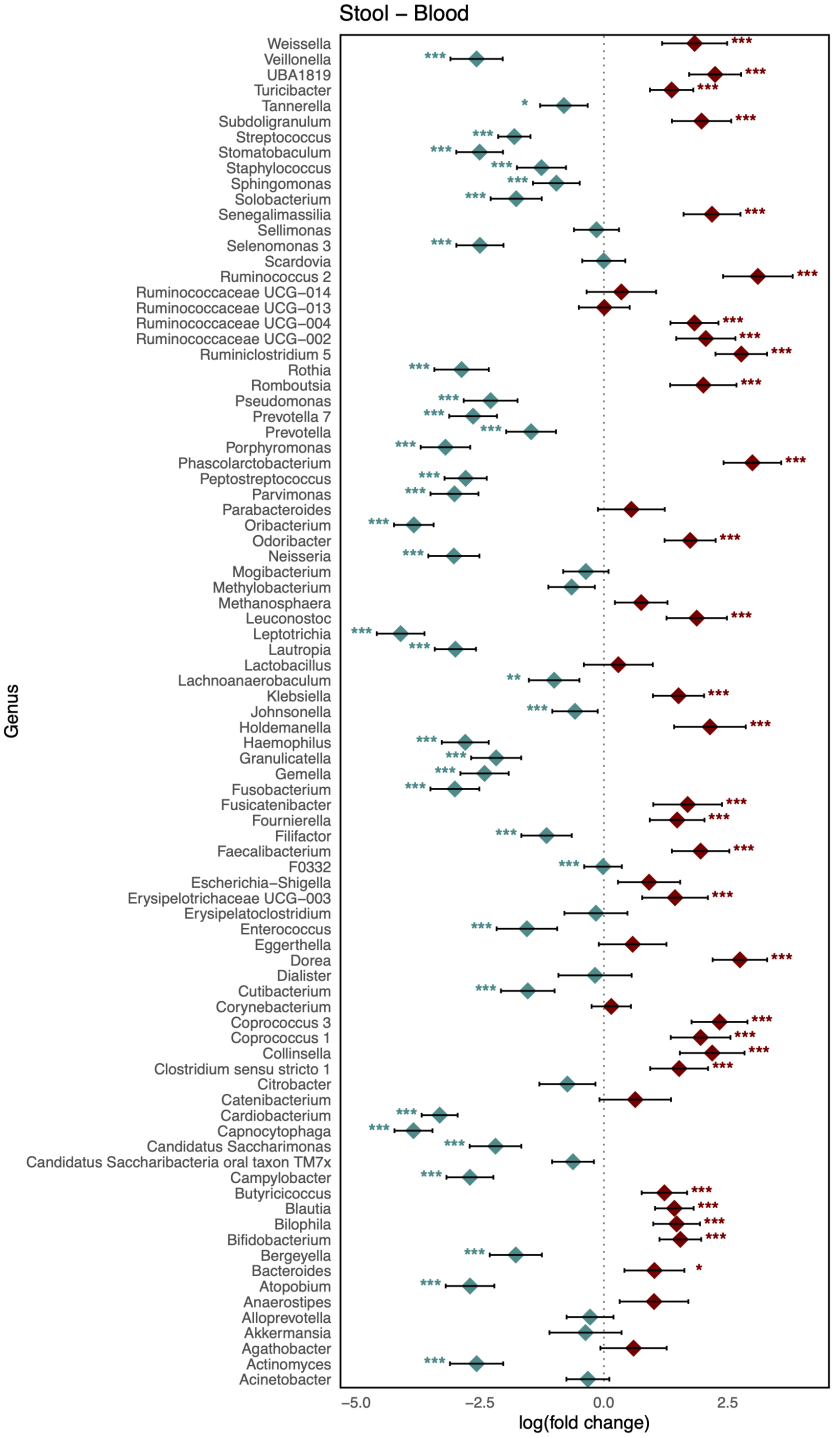
Log-scaled fold change (logFC) and 95% confidence interval (CI) of the abundance of each genus in stool vs. blood confirmed by differential abundance tests. Positive logFC values indicate a higher abundance in stool compared to blood, while negative values indicate a higher abundance in the latter. Points represent the estimated logFC for each genus, with red points indicating positive (higher abundance in stool) and blue points indicating negative (higher abundance in blood) value. Error bars represent the 95% CI for each estimate. *, false discovery rate (FDR) < 0.05; **, FDR < 0.01; ***, FDR < 0.001, denoting levels of statistical significance in abundance differences.

**Figure 5:**
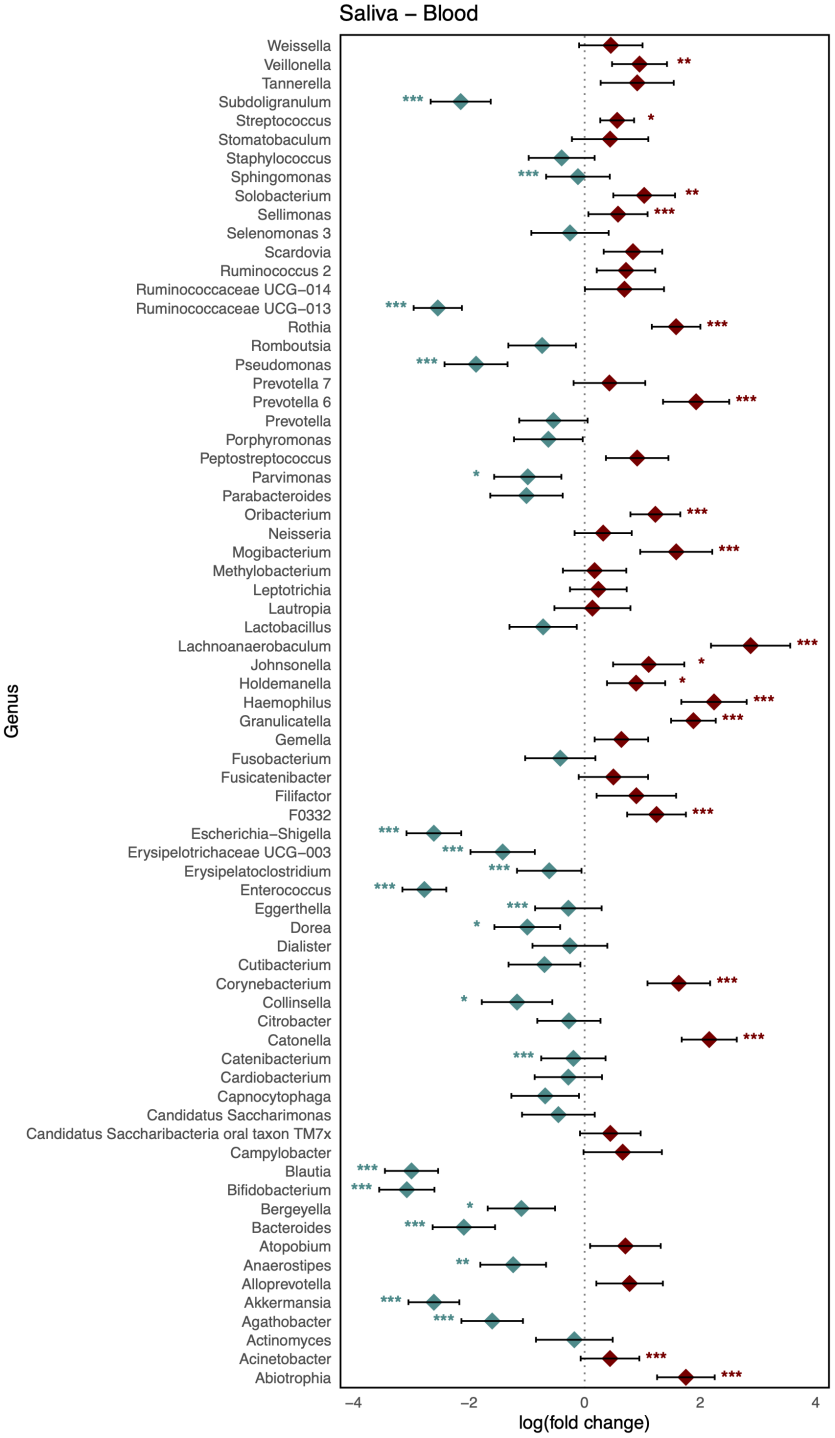
Log-scaled fold change (logFC) and 95% confidence interval (CI) of the abundance of each genus in saliva vs. blood confirmed by differential abundance tests. Positive logFC values indicate a higher abundance in saliva compared to blood, while negative values indicate a higher abundance in the latter. Points represent the estimated logFC for each genus, with red points indicating positive (higher abundance in saliva) and blue points indicating negative (higher abundance in blood) value. Error bars represent the 95% CI for each estimate. *, false discovery rate (FDR) < 0.05; **, FDR < 0.01; ***, FDR < 0.001, denoting levels of statistical significance in abundance differences.

As shown in **Figure 3**, comparison of the healthy microbiomes in stool and saliva revealed that the genera showing the greatest differences between the two habitats were *Bifidobacterium* and *Haemophilus* (logFC: 4.75 [95% CI: 4.06, 5.44] and −4.91 [−5.63, −4.19]; FDR < 0.001, respectively). In addition, 32 genera, including *Bacteroides*, *Bifidobacterium*, *Enterococcus*, and *Faecalibaterium* were enriched in stool compared to saliva, and another 32 genera, including *Veillonella*, *Streptococcus*, and *Rothia*, were enriched in saliva. Two species of *Ruminococcus* were highly abundant in stool (logFC: 3.11 [95% CI: 2.40, 3.81]; FDR < 0.001), whereas *Leptotrichia* was significantly enriched in blood (logFC: −4.10 [95% CI: −4.58, −3.61]; FDR < 0.001). Twenty-nine genera, including *Klebsiella*, *Faecalibacterium*, and *Bifidobacterium*, showed increased abundance in stool, and 36 genera, including *Veillonella*, *Streptococcus*, and *Staphylococcus*, showed increased abundance in blood (**Figure 4**). **Figure 5** shows the baseline abundance of the genera in saliva and blood. Eighteen genera, including *Acinetobacter*, *Haemophilus*, and *Rothia*, were enriched in saliva compared to blood, whereas 20 genera, including *Bifidobacterium*, *Bacteroides*, and *Pseudomonas*, were enriched in blood. The most differentially abundant genera were *Lachnoanaerobaculum* and *Bifidobacterium* (logFC: 2.87 [95% CI: 2.19, 3.56] and −3.08 [−3.56, −2.60], respectively).

### 3.4 Reference microbiome profiles representing disease-free status of healthy individuals

The microbiome profiles of healthy subjects were validated by comparison with those of the PD group to obtain a human reference microbiome profile representative of healthy microbial status, distinguishable from disease conditions. For all subjects in the PD group, Shannon index values for the saliva and stool microbiomes fell within the reference intervals, while only two subjects showed blood microbiome values outside the reference range. Other alpha diversity indices displayed similar patterns (**Supplementary Table S3**).

In terms of taxonomic profiles, for each of the two habitats in the PD group, we investigated whether the fold change (FC) in abundance of each taxon differed from healthy subjects. For the phylum-level abundance of stool and saliva microbiomes, the logFC of the abundance of Firmicutes, Patescibacteria, and Verrucucomicrobia had values outside the range of the reference group in all subjects in the PD group (**Table 2**). With regard to the FCs representing the abundance of phyla in the stool with respect to blood, Cyanobacteria, Fusobacteria, and Tenericutes in all subjects in the PD group showed distinct values from the healthy group (**Table 3**). Epsilonbacteraeota, Firmicutes, Fusobacteria, and Proteobacteria showed distinct values in all subjects in the PD group versus the healthy group in comparison of saliva and blood microbiomes (**Table 4**). The same process was repeated for genus-level abundance and FC values of 51, 51, and 39 genera in all subjects in the PD group were distinguished from those in the reference group for stool and saliva, stool and blood, and saliva and blood, respectively; these genera are listed in **Supplementary Tables S4**, **S5**, and **S6**. Moreover, PCA was performed on the logFC of the chosen taxa in stool and saliva, stool and blood, and saliva and blood, respectively. All three results show the separation between the reference and PD group (**Supplementary Figure S2**).

**Table 2:** 95% CIs of logFC representing the abundance of each phylum in the microbiome of stool vs. that of saliva in the reference group and proportions of subjects with PD that fell within the ranges of the reference group.

Phylum	logFC (stool/blood) (95% CI)	Proportion of PD group within range
Actinobacteria	−0.18 [−0.67, 0.31]	0.3
Bacteroidetes	0.03 [−0.57, 0.63]	0.6
Epsilonbacteraeota	−3.07 [−3.67, −2.47]	0.3
Firmicutes	0.07 [−0.35, 0.49]	0
Fusobacteria	−3.92 [−4.59, −3.26]	0.2
Patescibacteria	−1.89 [−2.58, −1.19]	0
Proteobacteria	−0.56 [−1.20, 0.07]	0.2
Tenericutes	0.80 [0.15, 1.44]	0.1
Verrucomicrobia	2.60 [1.76, 3.44]	0

95% CI, 95% confidence interval; logFC, log-scaled fold change; PD, periodontal disease.

**Table 3:** 95% CIs of logFC representing the abundance of each phylum in the microbiome of stool vs. that of blood in the reference group and proportions of subjects with PD that fell within the ranges of the reference group.

Phylum	logFC (stool/blood) (95% CI)	Proportion of PD group within range
Actinobacteria	0.81 [0.44, 1.17]	0.1
Bacteroidetes	0.07 [−0.39, 0.54]	0.6
Cyanobacteria	0.11 [−0.38, 0.61]	0
Epsilonbacteraeota	−2.51 [−3.03, −1.98]	0.1
Euryarchaeota	1.02 [0.37, 1.66]	0.8
Firmicutes	0.65 [0.33, 0.96]	0.1
Fusobacteria	−3.57 [−4.14, −3.01]	0
Patescibacteria	−2.31 [−2.69, −1.57]	0.2
Proteobacteria	−0.14 [−0.63, 0.34]	0.3
Tenericutes	2.22 [1.66, 2.77]	0
Verrucomicrobia	−0.18 [−0.94, 0.59]	0.1

95% CI, 95% confidence interval; logFC, log-scaled fold change; PD, periodontal disease.

**Table 4:** 95% CIs of logFC representing the abundance of each phylum in the microbiome of stool vs. that of blood in the reference group and proportions of subjects with PD that fell within the ranges of the reference group.

Phylum	logFC (saliva/blood) (95% CI)	Proportion of PD group within range
Actinobacteria	0.47 [0.17, 0.78]	0.2
Bacteroidetes	−0.47 [−0.82, −0.13]	0.3
Cyanobacteria	0.08 [−0.38, 0.53]	0.1
Epsilonbacteraeota	0.05 [−0.52, 0.62]	0
Firmicutes	0.06 [−0.18, 0.31]	0
Fusobacteria	−0.16 [−0.51, 0.18]	0
Patescibacteria	−0.75 [−1.19, −0.34]	0.2
Proteobacteria	−0.09 [−0.45, 0.28]	0
Verrucomicrobia	−3.29 [−3.68, −2.90]	0.1

95% CI, 95% confidence interval; logFC, log-scaled fold change; PD, periodontal disease.

## 4 Discussion

Laboratory testing is carried out to answer medical questions related to health risks, diagnosis, selection of treatment, and prognosis. The prerequisite of such testing is the equivalence between different measurements to ensure consistent interpretation of results and avoid harm to patients due to diagnostic errors (Plebani, 2013; Tate et al., 2014). As a comparison of results with reference or therapeutic ranges without harmonization can lead to misinterpretation, the Joint Committee for Traceability in Laboratory Medicine (JCTLM) was established to achieve standardization and global harmonization of clinical laboratory test results (Armbruster and Miller, 2007).

Quantitative medical laboratory tests should provide biological reference intervals or clinical decision limits to aid in the interpretation of the results. Regardless of the particular disease, the reference interval aims to identify 95% of the subpopulation likely to be disease-free (Jones and Barker, 2008; Ozarda, 2016; Haeckel et al., 2023). When deciding on any reference interval or clinically meaningful limits, it is important to consider factors that influence variations in measurements, intra- and interindividual variability, and analytical and preanalytical variability to ensure that it contains only nonpathological values of the measure of interest (Miller et al., 2023).

The microbiome is sensitive to the environment, and demographic or biological factors can cause interindividual variability of microbiome composition (Gerber, 2014; Wang and LeCao, 2020). In addition, technical factors in high-throughput sequencing, such as sampling and the sequencing batch, can result in uneven sequencing depth and may introduce unwanted variation and spurious heterogeneity into the data (Gilad et al., 2009; Boers et al., 2019; Wang and LeCao, 2020). Therefore, although several studies have suggested the potential of microbiome profiles as diagnostic or prognostic markers for several diseases (Persson and Persson, 2008; Amar et al., 2011; Rajendhran et al., 2013; Santiago et al., 2016; Dizdar et al., 2017; Meng et al., 2018; Dioguardi et al., 2020), there remains an unmet need for a standardized and fit-for-purpose approach to produce harmonized quantitative results with interpretable limits.

Several inherent characteristics of microbiome data hinder the application of traditional statistical methods, thereby making their clinical application difficult. Most importantly, microbiome compositions are quantified as sparse compositional vectors. That is, microbiome counts have an excess number of coordinates equal to zero and a constraint on the sum of the coordinates (Tsilimigras and Fodor, 2016; Gloor et al., 2017). The large number of zeros leads to the problem of discerning the real absence of microbiome species from undersampling. Sampling bias and uneven sequencing depth result in the compositionality of microbiome data that what we observe is the abundance as proportions with a unit sum and not the absolute number of microorganisms (Weiss et al., 2015; Tsilimigras and Fodor, 2016; Gloor et al., 2017). To overcome these limitations, it is necessary to apply statistical methods capable of specifically accounting for sparsity and compositionality, such as log-ratio data transformation or zero-inflated linear models, e.g., the Gaussian, negative binomial mixed, or Dirichlet multinomial models (Tsilimigras and Fodor, 2016; Dai et al., 2019; Wang and LeCao, 2020; Bharti and Grimm, 2021).

Considering the significance of reference values in laboratory medicine and the inherent limitations of microbiome data, we attempted to determine human microbiome reference profiles using blood, saliva, and stool samples from healthy subjects chosen according to predefined criteria. Diversity indices are quantitative indicators used in ecology to show how many organisms are distributed in a particular ecosystem and their evolutionary relevance (Ursell et al., 2012; Thukral, 2017). Although they are widely used in medical research to investigate changes in biodiversity due to the onset of disease or any intervention, there have been no attempts to establish meaningful limits capable of representing the microbial diversity of reference populations in the context of traditional laboratory medicine. Given the importance of age- or sex-stratified reference intervals for various clinical measures (Breiner et al., 2019; Bawua et al., 2022; Miller et al., 2023), we first examined the age- or sex-specific variability of microbiome diversity. Instead of beta-diversity indices representing the distance between two samples, the reference intervals of three alpha-diversity indices with one-to-one correspondence were calculated. Furthermore, we suggest another indicator for screening the disease status of microbiomes: the FC in the abundance of microorganisms residing in two habitats within the same subject. The indicator was drawn out using ANCOM-BC, a powerful DA algorithm that makes use of statistical methods to address the inherent limitations of microbiome data. This method uses expectation-maximization algorithms to estimate the unknown sampling fractions of each sample and therefore estimate the bias-corrected abundance of each taxon. In addition, the logFC values derived using linear regression correspond to log ratio transformations to handle the compositionality of microbiome data (Lin and Peddada, 2020). As it represents the degree of difference in the abundance of microbes between two specimens from a single individual collected at the same time, it could reduce the effects of intraindividual biological variability.

To determine the reference microbiome profile, we examined whether the microbial diversity and taxonomic profiles of the reference group could distinguish the microbial status of subjects in the PD group from healthy controls. With well-balanced baseline characteristics between the reference and PD groups (**Table 1**), any observed differences can be attributed specifically to disease status rather than demographic variability. Although alpha diversity indices themselves could not differentiate the PD group from the reference group, we identified several microbial taxa where the FC in abundance between two habitats in PD subjects exceeded the range observed in the reference group.

For stool and saliva microbiomes, the abundance of Firmicutes, Patescibacteria, and Verrucucomicrobia could effectively distinguish subjects with PD from healthy individuals. That is, the differences between their abundances in the two habitats for healthy individuals fell within the 95% CI of logFC [−0.18, 0.31], [−1.19, −0.34], and [−3.68, −2.90], respectively. The differences in abundance of Cyanobacteria, Fusobacteria, and Tenericutes in stool and blood microbiomes of the healthy subjects fell within the 95% CI of logFC [−0.38, 0.61], [−4.14, −3.01], and [1.66, 2.77], respectively. When saliva and blood microbiome of a subject is analyzed, the differences in abundance of Epsilonbacteraeota, Firmicutes, Fusobacteria, and Proteobacteria would be useful to determine healthy status (95% CI of logFC: [−3.67, −2.47], [−0.35, 0.49], [−4.59, −3.26], and [−1.20, 0.07], respectively). As differences in these values were even discernible between healthy controls and individuals with PD, a relatively mild disease state, they can be applied as reference values representing the healthy status of the microbiome and be used to screen for microbiome alterations in disease states, preferably in preclinical stages.

Just as clinical laboratories require two different matched samples for several next-generation sequencing-based tests, e.g., tumor tissue with matched normal blood samples, we believe collecting two types of samples for microbiome profiling is a feasible scenario. Since blood is the most common specimen used in a majority of medical examinations, including routine checkups or acute visits, blood would be the simplest option to choose. The remaining one would be chosen between saliva and stool considering its association with the disease of interest and convenience and repeatability of sampling.

Despite the diagnostic potential of reference microbiome profiles, further studies involving larger and more diverse cohorts are essential to validate their biological and clinical significance. In particular, incorporating subjects with a wide range of systemic diseases, such as cardiometabolic disorders, chronic inflammatory conditions, and various types of cancer is crucial to evaluate the specificity and robustness of the reference profiles. The approach proposed in this study should also be expanded to populations of different races, ethnicities, and geographic regions to ensure the generalizability of reference values. The influence of genetic predispositions, dietary habits, and environmental exposures on microbiome composition necessitates a more comprehensive understanding to determine global reference intervals. Furthermore, although our study found no or limited associations between microbial diversity and clinical variables such as BMI, alcohol consumption, and tobacco smoking, previous research has demonstrated the influence of these factors, particularly on gut and oral microbiomes (Capurso and Lahner, 2017; Maruvada et al., 2017; Benahmed et al., 2021; Huang and Shi, 2019). These findings underscore the need for more precise, detailed examination of lifestyle factors, as our study relied primarily on self-reported questionnaires and binary categorical responses (e.g., yes or no). Future studies should incorporate comprehensive, quantitative lifestyle data to further stratify reference profiles based on microbiome-influencing factors beyond traditional age- and sex-based intervals. Additionally, despite employing the advanced statistical model ANCOM-BC, the FC approach retains inherent limitations, particularly for low-abundance taxa. Specifically, taxa present at very low levels may exhibit disproportionately large fold-change values due to high variability and fluctuations near the detection limit, which are unlikely to represent biologically meaningful differences. To address this issue, validation through complementary methods, such as PCR-based absolute quantification, could improve the accuracy and reliability of the reference values. Such refinements would enhance the utility of microbiome reference profiles, providing more accurate baselines for diagnostic and research applications. The reference microbiome profiles for differentiating pathological conditions of the human microbiome from the healthy status will expand the scope of laboratory medicine and facilitate the development of new diagnostic and disease-monitoring strategies.

## Data Availability

The datasets presented in this study can be found in online repositories. The names of the repository/repositories and accession number(s) can be found below: https://www.ncbi.nlm.nih.gov/, PRJNA1027093.
