# Supplementary material for: Human reference microbiome profiles of different body habitats in healthy individuals
Source: Front Cell Infect Microbiol. 2025 Feb 11;15:1478136. doi: 10.3389/fcimb.2025.1478136 (PMC11850547; doi:10.3389/fcimb.2025.1478136)
Supplement: Supplementary file 1 [file DataSheet1.docx]

Supplementary Material

**Supplementary Figure 1.** Alpha diversity analysis of the blood, saliva, and stool microbiomes by categorical clinical variables. Box plots represent each diversity index stratified by clinical variables: sex (M/F) at the top, alcohol consumption (yes/no) in the middle, and smoking status (yes/no) at the bottom. The statistical significance of alpha diversity differences across these variables was assessed using the Wilcoxon rank-sum test (*P < 0.05; **P < 0.01; NS, not significant).

**Supplementary Figure 2.** Alpha diversity analysis of blood, saliva, and stool microbiomes in association with continuous clinical variables. Scatter plots show the trend of each diversity index against continuous variables: age at the top and body mass index (BMI) at the bottom. A trend line indicates the direction of association. Spearman correlation tests were used to evaluate correlations, with the correlation coefficient and significance level displayed in the top right corner of each plot.

**Supplementary Figure 3.** Biplot representations of the reference microbiome profiles. Principal component analysis was performed on the log-scaled fold change values of the abundance of taxa in stool vs. saliva, stool vs. blood, and saliva vs. blood, respectively. The loading plot (arrows) indicates the contributions of phyla chosen as reference microbiome on the corresponding coordinates.

**Supplementary Table 1.** Microbial variations in blood, saliva, and stool associated with sex and lifestyle factors, including alcohol consumption and tobacco smoking, in reference group subjects. The strengths of associations between clinical variables and beta diversity indices were confirmed using the permutation multivariate analysis of variance (PERMANOVA) test.

| Habitat | Variable | Diversity Index | R^2^ | P-value |
| --- | --- | --- | --- | --- |
| Blood | Sex (M/F) | Bray-Curtis | 0.016 | 0.014 |
|  |  | Unweighted UniFrac | 0.012 | 0.095 |
| Saliva |  | Bray-Curtis | 0.009 | 0.217 |
|  |  | Unweighted UniFrac | 0.009 | 0.312 |
| Stool |  | Bray-Curtis | 0.011 | 0.345 |
|  |  | Unweighted UniFrac | 0.012 | 0.302 |
| Blood | Alcohol consumption (yes/no) | Bray-Curtis | 0.034 | 0.001 |
|  |  | Unweighted UniFrac | 0.020 | 0.002 |
| Saliva |  | Bray-Curtis | 0.010 | 0.109 |
|  |  | Unweighted UniFrac | 0.012 | 0.111 |
| Stool |  | Bray-Curtis | 0.010 | 0.438 |
|  |  | Unweighted UniFrac | 0.014 | 0.087 |
| Blood | Smoking habit (yes/no) | Bray-Curtis | 0.008 | 0.380 |
|  |  | Unweighted UniFrac | 0.008 | 0.533 |
| Saliva |  | Bray-Curtis | 0.008 | 0.169 |
|  |  | Unweighted UniFrac | 0.008 | 0.137 |
| Stool |  | Bray-Curtis | 0.013 | 0.684 |
|  |  | Unweighted UniFrac | 0.013 | 0.428 |

**Supplementary Table 2.** Microbial variations in blood, saliva, and stool associated with age and BMI of reference group subjects. Associations between clinical variables and beta diversity indices were confirmed by the Mantel test using Spearman’s correlation.

| Habitat | Variable | Diversity Index | Spearman’s Coefficient (rho) | P-value |
| --- | --- | --- | --- | --- |
| Blood | Age (yrs) | Bray-Curtis | 0.020 | 0.650 |
|  |  | Unweighted UniFrac | -0.030 | 0.410 |
| Saliva |  | Bray-Curtis | 0.080 | 0.010 |
|  |  | Unweighted UniFrac | 0.020 | 0.500 |
| Stool |  | Bray-Curtis | 0.010 | 0.830 |
|  |  | Unweighted UniFrac | -0.020 | 0.630 |
| Blood | BMI (kg/m^2^) | Bray-Curtis | -0.033 | 0.846 |
|  |  | Unweighted UniFrac | -0.044 | 0.749 |
| Saliva |  | Bray-Curtis | -0.017 | 0.634 |
|  |  | Unweighted UniFrac | 0.030 | 0.312 |
| Stool |  | Bray-Curtis | -0.005 | 0.519 |
|  |  | Unweighted UniFrac | 0.022 | 0.320 |

**Supplementary Table 3.** Reference intervals of alpha diversity indices calculated as values of central 95% of the reference group and proportions of subjects with PD that fell within the reference range.

| Habitat | Alpha Diversity Index | Refence intervals | Proportion of PD group within range |
| --- | --- | --- | --- |
| Blood | Shannon's entropy | 7.214–8.157 | 0.8 |
|  | Simpson's index | 0.990–0.995 | 0.8 |
|  | Pielou's evenness | 0.897–0.947 | 0.8 |
| Saliva | Shannon's entropy | 5.581–7.692 | 1 |
|  | Simpson's index | 0.973–0.993 | 1 |
|  | Pielou's evenness | 0.853–0.949 | 1 |
| Stool | Shannon's entropy | 5.050–7.599 | 1 |
|  | Simpson's index | 0.961–0.993 | 1 |
|  | Pielou's evenness | 0.755–0.942 | 1 |

Abbreviations: PD, periodontal disease.

**Supplementary Table 4.** 95% CIs of logFC representing the abundance of each genus in the microbiome of stool vs. that of saliva in the reference group and proportions of subjects with PD that fell within the ranges of the reference group.

| Genus | logFC (stool/saliva) (95% CI) | | Proportion of PD group within range |
| --- | --- | --- | --- |
| *Actinomyces* | -2.26 [-3.03, -1.49] | 0 | |
| *Agathobacter* | 2.33 [1.60, 3.06] | 0 | |
| *Akkermansia* | 2.37 [1.47, 3.26] | 0 | |
| *Anaerostipes* | 2.38 [1.64, 3.11] | 0.1 | |
| *Atopobium* | -3.28 [-4.01, -2.55] | 0.4 | |
| *Bacteroides* | 3.24 [2.41, 4.06] | 0.1 | |
| *Barnesiella* | -0.44 [1.00, -1.04] | 0 | |
| *Bifidobacterium* | 4.75 [4.06, 5.44] | 0.5 | |
| *Bilophila* | 0.16 [1.00, -0.45] | 0 | |
| *Blautia* | 4.55 [3.94, 5.16] | 0.1 | |
| *Butyricicoccus* | -0.17 [1.00, -0.77] | 0 | |
| *Campylobacter* | -3.24 [-3.90, -2.57] | 0.1 | |
| *Candidatus Saccharimonas* | -1.61 [-2.27, -0.94] | 0 | |
| *Capnocytophaga* | -3.03 [-3.76, -2.30] | 0 | |
| *Cardiobacterium* | -2.90 [-3.55, -2.26] | 0.3 | |
| *Catenibacterium* | 0.95 [0.18, 1.72] | 0.3 | |
| *Catonella* | -1.51 [-2.04, -0.98] | 0 | |
| *Clostridium sensu stricto 1* | 0.63 [-0.06, 1.31] | 0.2 | |
| *Collinsella* | 3.48 [2.77, 4.19] | 0 | |
| *Coprococcus 1* | 1.20 [0.50, 1.90] | 0 | |
| *Coprococcus 3* | 1.12 [0.42, 1.83] | 0 | |
| *Corynebacterium* | -1.35 [-1.90, -0.81] | 0 | |
| *Dialister* | 0.20 [1.00, -0.63] | 0 | |
| *Dorea* | 3.86 [3.28, 4.45] | 0.3 | |
| *Eggerthella* | 0.99 [0.28, 1.69] | 0.2 | |
| *Enterococcus* | 1.35 [0.59, 2.10] | 0 | |
| *Erysipelatoclostridium* | 0.58 [-0.08, 1.24] | 0.6 | |
| *Erysipelotrichaceae UCG-003* | 2.98 [2.26, 3.71] | 0 | |
| *Escherichia-Shigella* | 3.65 [2.85, 4.45] | 0 | |
| *Faecalibacterium* | 0.90 [0.20, 1.60] | 0 | |
| *Filifactor* | -1.93 [-2.54, -1.31] | 0 | |
| *Fournierella* | 0.55 [-0.10, 1.19] | 0.6 | |
| *Fusicatenibacter* | 1.32 [0.55, 2.09] | 0.1 | |
| *Fusobacterium* | -2.46 [-3.27, -1.65] | 0 | |
| *Gemella* | -2.92 [-3.65, -2.19] | 0 | |
| *Granulicatella* | -3.93 [-4.62, -3.24] | 0.4 | |
| *Haemophilus* | -4.91 [-5.63, -4.19] | 0 | |
| *Holdemanella* | 1.38 [0.55, 2.20] | 0 | |
| *Johnsonella* | -1.56 [-2.12, -1.01] | 0 | |
| *Lachnoanaerobaculum* | -3.75 [-4.38, -3.12] | 0.3 | |
| *Lactobacillus* | 1.14 [0.06, 0.39] | 0 | |
| *Lautropia* | -3.00 [-3.68, -2.33] | 0.1 | |
| *Leptotrichia* | -4.21 [-4.96, -3.46] | 0.5 | |
| *Leuconostoc* | 1.04 [0.08, 0.34] | 0.4 | |
| *Mogibacterium* | -1.82 [-2.41, -1.23] | 0 | |
| *Neisseria* | -3.22 [-4.00, -2.44] | 0 | |
| *Odoribacter* | 0.57 [-0.08, 1.22] | 0.1 | |
| *Oribacterium* | -4.93 [-5.57, -4.30] | 0.1 | |
| *Parabacteroides* | 1.69 [0.92, 2.46] | 0 | |
| *Parvimonas* | -1.90 [-2.70, -1.11] | 0.2 | |
| *Peptostreptococcus* | -3.57 [-4.29, -2.85] | 0.3 | |
| *Phascolarctobacterium* | 1.48 [0.74, 2.21] | 0 | |
| *Porphyromonas* | -2.44 [-3.28, -1.61] | 0.1 | |
| *Prevotella 6* | -1.78 [-2.35, -1.21] | 0 | |
| *Prevotella 7* | -2.94 [-3.72, -2.16] | 0 | |
| *Romboutsia* | 2.87 [2.15, 3.58] | 0.1 | |
| *Rothia* | -4.33 [-5.09, -3.57] | 0.6 | |
| *Ruminiclostridium 5* | 1.43 [0.75, 2.10] | 0 | |
| *Ruminococcaceae UCG-002* | 1.16 [0.46, 1.85] | 0 | |
| *Ruminococcaceae UCG-004* | 0.34 [1.00, -0.31] | 0 | |
| *Ruminococcaceae UCG-013* | 2.68 [1.98, 3.38] | 0.4 | |
| *Ruminococcaceae UCG-014* | -0.21 [1.00, -1.05] | 0 | |
| *Ruminococcus 2* | 2.52 [1.73, 3.31] | 0 | |
| *Selenomonas 3* | -2.12 [-2.76, -1.48] | 0 | |
| *Solobacterium* | -2.67 [-3.44, -1.90] | 0.3 | |
| *Stomatobaculum* | -2.82 [-3.46, -2.18] | 0.2 | |
| *Streptococcus* | -2.24 [-2.75, -1.74] | 0.1 | |
| *Subdoligranulum* | 4.24 [3.45, 5.04] | 0.7 | |
| *Tannerella* | -1.59 [-2.17, -1.01] | 0 | |
| *UBA1819* | 0.89 [0.21, 1.57] | 0.3 | |
| *Veillonella* | -3.39 [-4.17, -2.62] | 0.1 | |
| *Weissella* | 1.50 [0.78, 2.22] | 0 | |

Abbreviations: 95% CI, 95% confidence interval; logFC, log-scaled fold change; PD, periodontal disease.

**Supplementary Table 5.** 95% CIs of logFC representing the abundance of each genus in the microbiome of stool vs. that of blood in the reference group and proportions of subjects with PD that fell within the ranges of the reference group.

| Genus | logFC (stool/blood) (95% CI) | Proportion of PD group within range |
| --- | --- | --- |
| *Acinetobacter* | -0.32 [-0.76, 0.11] | 0.1 |
| *Actinomyces* | -2.57 [-3.11, -2.03] | 0 |
| *Agathobacter* | 0.60 [-0.07, 1.27] | 0.2 |
| *Akkermansia* | -0.37 [-1.10, 0.36] | 0.6 |
| *Alloprevotella* | -0.28 [-0.75, 0.19] | 0.2 |
| *Anaerostipes* | 1.01 [0.32, 1.70] | 0 |
| *Atopobium* | -2.70 [-3.19, -2.21] | 0 |
| *Bacteroides* | 1.02 [0.41, 1.63] | 0.3 |
| *Bergeyella* | -1.78 [-2.30, -1.25] | 0 |
| *Bifidobacterium* | 1.54 [1.12, 1.96] | 0 |
| *Bilophila* | 1.47 [1.00, 1.94] | 0 |
| *Blautia* | 1.42 [1.03, 1.81] | 0.2 |
| *Butyricicoccus* | 1.22 [0.76, 1.68] | 0.1 |
| *Campylobacter* | -2.70 [-3.18, -2.23] | 0.1 |
| *Candidatus Saccharimonas* | -2.19 [-2.71, -1.67] | 0 |
| *Capnocytophaga* | -3.84 [-4.23, -3.46] | 0 |
| *Cardiobacterium* | -3.31 [-3.68, -2.95] | 0 |
| *Catenibacterium* | 0.63 [-0.09, 1.36] | 0.5 |
| *Citrobacter* | -0.74 [-1.30, -0.17] | 0.3 |
| *Clostridium sensu stricto 1* | 1.52 [0.94, 2.10] | 0 |
| *Collinsella* | 2.19 [1.53, 2.84] | 0 |
| *Coprococcus 1* | 1.95 [1.35, 2.55] | 0 |
| *Coprococcus 3* | 2.33 [1.77, 2.90] | 0 |
| *Corynebacterium* | 0.15 [-0.25, 0.55] | 0.6 |
| *Cutibacterium* | -1.54 [-2.08, -0.99] | 0 |
| *Dialister* | -0.18 [-0.92, 0.56] | 0.6 |
| *Dorea* | 2.75 [2.20, 3.29] | 0 |
| *Eggerthella* | 0.58 [-0.10, 1.26] | 0.6 |
| *Enterococcus* | -1.55 [-2.16, -0.94] | 0.1 |
| *Erysipelatoclostridium* | -0.16 [-0.80, 0.48] | 0.7 |
| *Erysipelotrichaceae UCG-003* | 1.44 [0.77, 2.10] | 0 |
| *Escherichia-Shigella* | 0.91 [0.29, 1.54] | 0.2 |
| *F0332* | -0.02 [-0.40, 0.36] | 0.8 |
| *Faecalibacterium* | 1.95 [1.37, 2.53] | 0 |
| *Filifactor* | -1.16 [-1.67, -0.65] | 0 |
| *Fournierella* | 1.48 [0.93, 2.03] | 0 |
| *Fusicatenibacter* | 1.69 [1.00, 2.38] | 0.1 |
| *Fusobacterium* | -3.01 [-3.50, -2.51] | 0 |
| *Gemella* | -2.41 [-2.89, -1.92] | 0 |
| *Granulicatella* | -2.17 [-2.68, -1.67] | 0 |
| *Haemophilus* | -2.80 [-3.27, -2.32] | 0.2 |
| *Holdemanella* | 2.14 [1.42, 2.86] | 0 |
| *Johnsonella* | -0.58 [-1.04, -0.12] | 0.3 |
| *Klebsiella* | 1.51 [0.99, 2.02] | 0 |
| *Lachnoanaerobaculum* | -1.00 [-1.51, -0.49] | 0 |
| *Lactobacillus* | 0.29 [-0.40, 0.99] | 0 |
| *Lautropia* | -3.00 [-3.41, -2.58] | 0 |
| *Leptotrichia* | -4.10 [-4.58, -3.62] | 0 |
| *Leuconostoc* | 1.87 [1.26, 2.48] | 0 |
| *Methylobacterium* | -0.65 [-1.12, -0.18] | 0.3 |
| *Mogibacterium* | -0.36 [-0.82, 0.09] | 0.6 |
| *Neisseria* | -3.03 [-3.54, -2.51] | 0 |
| *Odoribacter* | 1.74 [1.22, 2.26] | 0 |
| *Oribacterium* | -3.83 [-4.23, -3.43] | 0.1 |
| *Parabacteroides* | 0.55 [-0.12, 1.23] | 0.2 |
| *Parvimonas* | -3.01 [-3.50, -2.53] | 0 |
| *Peptostreptococcus* | -2.79 [-3.22, -2.36] | 0 |
| *Phascolarctobacterium* | 3.00 [2.42, 3.58] | 0 |
| *Porphyromonas* | -3.20 [-3.70, -2.70] | 0 |
| *Prevotella* | -1.47 [-1.97, -0.97] | 0 |
| *Prevotella 7* | -2.64 [-3.12, -2.16] | 0 |
| *Pseudomonas* | -2.29 [-2.83, -1.74] | 0 |
| *Romboutsia* | 2.01 [1.34, 2.68] | 0 |
| *Rothia* | -2.87 [-3.42, -2.32] | 0 |
| *Ruminiclostridium 5* | 2.77 [2.25, 3.29] | 0.1 |
| *Ruminococcaceae UCG-002* | 2.06 [1.46, 2.65] | 0 |
| *Ruminococcaceae UCG-004* | 1.83 [1.34, 2.31] | 0 |
| *Ruminococcaceae UCG-013* | 0.01 [-0.50, 0.52] | 0 |
| *Ruminococcaceae UCG-014* | 0.35 [-0.35, 1.05] | 0.3 |
| *Ruminococcus 2* | 3.11 [2.40, 3.81] | 0.2 |
| *Scardovia* | 0.00 [-0.44, 0.43] | 0.8 |
| *Selenomonas 3* | -2.50 [-2.98, -2.03] | 0 |
| *Solobacterium* | -1.77 [-2.28, -1.25] | 0 |
| *Sphingomonas* | -0.96 [-1.43, -0.49] | 0 |
| *Staphylococcus* | -1.26 [-1.75, -0.76] | 0 |
| *Stomatobaculum* | -2.51 [-2.98, -2.04] | 0 |
| *Streptococcus* | -1.81 [-2.13, -1.48] | 0.1 |
| *Subdoligranulum* | 1.97 [1.37, 2.57] | 0 |
| *Tannerella* | -0.81 [-1.29, -0.33] | 0.1 |
| *Turicibacter* | 1.37 [0.93, 1.80] | 0 |
| *UBA1819* | 2.24 [1.72, 2.77] | 0 |
| *Veillonella* | -2.57 [-3.10, -2.04] | 0.1 |
| *Weissella* | 1.83 [1.17, 2.49] | 0.1 |

Abbreviations: 95% CI, 95% confidence interval; logFC, log-scaled fold change; PD, periodontal disease.

**Supplementary Table 6.** 95% CIs of logFC representing the abundance of each genus in the microbiome of saliva vs. that of blood in the reference group and proportions of subjects with PD that fell within the ranges of the reference group.

| Genus | logFC (saliva/blood) (95% CI) | Proportion of PD group within range |
| --- | --- | --- |
| *Acinetobacter* | 0.44 [-0.07, 0.95] | 0.2 |
| *Actinomyces* | -0.18 [1.00, -0.84] | 0 |
| *Agathobacter* | -1.60 [-2.13, -1.07] | 0 |
| *Akkermansia* | -2.61 [-3.05, -2.17] | 0 |
| *Alloprevotella* | 0.78 [0.34, 0.20] | 0 |
| *Anaerostipes* | -1.24 [-1.81, -0.67] | 0 |
| *Atopobium* | 0.71 [0.80, 0.09] | 0 |
| *Bacteroides* | -2.09 [-2.63, -1.55] | 0 |
| *Bergeyella* | -1.10 [-1.68, -0.51] | 0 |
| *Bifidobacterium* | -3.08 [-3.56, -2.60] | 0 |
| *Blautia* | -3.00 [-3.46, -2.54] | 0 |
| *Campylobacter* | 0.66 [1.00, -0.02] | 0 |
| *Candidatus Saccharimonas* | -0.45 [1.00, -1.08] | 0 |
| *Capnocytophaga* | -0.68 [0.78, -1.27] | 0 |
| *Cardiobacterium* | -0.28 [1.00, -0.87] | 0 |
| *Catenibacterium* | -0.19 [-0.75, 0.36] | 0.9 |
| *Catonella* | 2.16 [1.68, 2.63] | 0 |
| *Citrobacter* | -0.27 [1.00, -0.82] | 0 |
| *Collinsella* | -1.17 [-1.78, -0.56] | 0 |
| *Corynebacterium* | 1.63 [1.09, 2.17] | 0.1 |
| *Cutibacterium* | -0.70 [0.88, -1.32] | 0 |
| *Dialister* | -0.26 [1.00, -0.90] | 0 |
| *Dorea* | -0.99 [-1.56, -0.42] | 0.1 |
| *Eggerthella* | -0.28 [-0.86, 0.29] | 0.9 |
| *Enterococcus* | -2.77 [-3.15, -2.39] | 0 |
| *Erysipelatoclostridium* | -0.61 [-1.17, -0.05] | 0.3 |
| *Erysipelotrichaceae UCG-003* | -1.42 [-1.98, -0.86] | 0 |
| *Escherichia-Shigella* | -2.61 [-3.08, -2.14] | 0 |
| *F0332* | 1.24 [0.74, 1.75] | 0.1 |
| *Filifactor* | 0.89 [0.45, 0.21] | 0 |
| *Fusicatenibacter* | 0.50 [1.00, -0.10] | 0 |
| *Fusobacterium* | -0.42 [1.00, -1.03] | 0 |
| *Gemella* | 0.64 [0.31, 0.17] | 0 |
| *Granulicatella* | 1.88 [1.50, 2.27] | 0 |
| *Haemophilus* | 2.24 [1.67, 2.80] | 0 |
| *Holdemanella* | 0.89 [0.39, 1.39] | 0.1 |
| *Johnsonella* | 1.11 [0.49, 1.72] | 0.1 |
| *Lachnoanaerobaculum* | 2.87 [2.19, 3.56] | 0.5 |
| *Lactobacillus* | -0.72 [0.59, -1.30] | 0 |
| *Lautropia* | 0.13 [1.00, -0.52] | 0 |
| *Leptotrichia* | 0.24 [1.00, -0.25] | 0 |
| *Methylobacterium* | 0.17 [1.00, -0.38] | 0 |
| *Mogibacterium* | 1.59 [0.96, 2.21] | 0 |
| *Neisseria* | 0.32 [1.00, -0.17] | 0 |
| *Oribacterium* | 1.22 [0.79, 1.66] | 0 |
| *Parabacteroides* | -1.01 [0.08, -1.63] | 0 |
| *Parvimonas* | -0.98 [-1.57, -0.40] | 0.1 |
| *Peptostreptococcus* | 0.91 [0.05, 0.37] | 0.2 |
| *Porphyromonas* | -0.63 [1.00, -1.22] | 0 |
| *Prevotella* | -0.54 [1.00, -1.13] | 0 |
| *Prevotella 6* | 1.93 [1.36, 2.50] | 0 |
| *Prevotella 7* | 0.43 [1.00, -0.19] | 0 |
| *Pseudomonas* | -1.88 [-2.42, -1.33] | 0 |
| *Romboutsia* | -0.74 [0.53, -1.32] | 0 |
| *Rothia* | 1.58 [1.16, 2.00] | 0 |
| *Ruminococcaceae UCG-013* | -2.54 [-2.96, -2.12] | 0 |
| *Ruminococcaceae UCG-014* | 0.69 [1.00, 0.00] | 0 |
| *Ruminococcus 2* | 0.72 [0.26, 0.21] | 0 |
| *Scardovia* | 0.84 [0.06, 0.33] | 0.3 |
| *Selenomonas 3* | -0.25 [1.00, -0.92] | 0 |
| *Solobacterium* | 1.03 [0.50, 1.57] | 0.1 |
| *Sphingomonas* | -0.12 [-0.67, 0.44] | 0.6 |
| *Staphylococcus* | -0.40 [1.00, -0.97] | 0 |
| *Stomatobaculum* | 0.44 [1.00, -0.22] | 0 |
| *Streptococcus* | 0.56 [0.27, 0.86] | 0.1 |
| *Subdoligranulum* | -2.15 [-2.67, -1.63] | 0 |
| *Tannerella* | 0.91 [0.23, 0.28] | 0.1 |
| *Veillonella* | 0.95 [0.47, 1.43] | 0.1 |
| *Weissella* | 0.45 [1.00, -0.10] | 0 |

Abbreviations: 95% CI, 95% confidence interval; logFC, log-scaled fold change; PD, periodontal disease.
